# Narrative and Pictorial Review on State-of-the-Art Endovascular Treatment for Focal Non-Infected Lesions of the Abdominal Aorta: Anatomical Challenges, Technical Solutions, and Clinical Outcomes

**DOI:** 10.3390/jcm14134798

**Published:** 2025-07-07

**Authors:** Mario D’Oria, Marta Ascione, Paolo Spath, Gabriele Piffaretti, Enrico Gallitto, Wassim Mansour, Antonino Maria Logiacco, Giovanni Badalamenti, Antonio Cappiello, Giulia Moretti, Luca Di Marzo, Gianluca Faggioli, Mauro Gargiulo, Sandro Lepidi

**Affiliations:** 1 Division of Vascular and Endovascular Surgery, Department of Medical Surgical and Health Sciences, University of Trieste, Strada di Fiume 447, 34149 Trieste, Italygiovanni.badalamenti@asugi.sanita.fvg.it (G.B.);; 2 Vascular and Endovascular Surgery Division, Department of General Surgery and Surgical Specialties, Policlinico Umberto I, “Sapienza” University of Rome, Viale del Policlinico 155, 00161 Rome, Italy; marta.ascione@uniroma1.it (M.A.); wassim.mansour@uniroma1.it (W.M.);; 3 Vascular Surgery, Department of Medical and Surgical Sciences, University of Bologna, 40138 Bologna, Italy; paolo.spath@gmail.com (P.S.);; 4 Vascular Surgery Department of Medicine and Surgery, School of Medicine, University of Insubria, 21100 Varese, Italy; gabriele.piffaretti@uninsubria.it

**Keywords:** aortic disease, endovascular repair, vascular surgery, narrative review

## Abstract

The natural history of focal non-infected lesions of the abdominal aorta (fl-AA) remains unclear and largely depends on their aetiology. These lesions often involve a focal “tear” or partial disruption of the arterial wall. Penetrating aortic ulcers (PAUs) and intramural hematomas (IMHs) are examples of focal tears in the aortic wall that can either progress to dilatation (saccular aneurysm) or fail to fully propagate through the medial layers, potentially leading to aortic dissection. These conditions typically exhibit a morphology consistent with eccentric saccular aneurysms. The management of focal non-infected pathologies of the abdominal aorta remains a subject of debate. Unlike fusiform abdominal aortic aneurysms, the inconsistent definitions and limited information regarding the natural history of saccular aneurysms (sa-AAAs) have prevented the establishment of universally accepted practice guidelines for their management. As emphasized in the latest 2024 ESVS guidelines, the focal nature of these diseases makes them ideal candidates for endovascular repair (class of evidence IIa—level C). Moreover, the Society for Vascular Surgery just referred to aneurysm diameter as an indication for treatment suggesting using a smaller diameter compared to fusiform aneurysms. Consequently, the management of saccular aneurysms is likely heterogeneous amongst different centres and different operators. Endovascular repair using tube stent grafts offers benefits like reduced recovery times but carries risks of migration and endoleak due to graft rigidity. These complications can influence long-term success. In this context, the use of endovascular bifurcated grafts may provide a more effective solution for treating these focal aortic pathologies. It is essential to achieve optimal sealing regions through anatomical studies of aortic morphology. Additionally, understanding the anatomical characteristics of focal lesions in challenging necks or para-visceral locations is indeed crucial in device choice. Off-the-shelf devices are favoured for their time and cost efficiency, but new endovascular technologies like fenestrated endovascular aneurysm repair (FEVAR) and custom-made devices enhance treatment success and patient safety. These innovations provide stent grafts in various lengths and diameters, accommodating different aortic anatomies and reducing the risk of type III endoleaks. Although complicated PAUs and focal saccular aneurysms rarely arise in the para-visceral aorta, the consequences of rupture in this segment might be extremely severe. Experience borrowed from complex abdominal and thoracoabdominal aneurysm repair demonstrates that fenestrated and branched devices can be deployed safely when anatomical criteria are respected. Elective patients derive the greatest benefit from a fenestrated graft, while urgent cases can be treated confidently with off-the-shelf multibranch systems, reserving other types of repairs for emergent or bail-out cases. While early outcomes of these interventions are promising, it is crucial to acknowledge that limited aortic coverage can still impede effective symptom relief and lead to complications such as aneurysm expansion or rupture. Therefore, further long-term studies are essential to consolidate the technical results and evaluate the durability of various graft options.

## 1. Introduction: Aetiology, Natural History and Morphology

The natural history of focal non-infected lesions of the abdominal aorta (fl-AA) remains unclear and largely depends on their aetiology. These lesions often involve a focal “tear” or partial disruption of the arterial wall. Penetrating aortic ulcers (PAU) and intramural hematomas (IMH) are examples of focal tears in the aortic wall that can either progress to dilatation (saccular aneurysm) or fail to fully propagate through the medial layers, potentially leading to aortic dissection. These conditions typically exhibit a morphology consistent with eccentric saccular aneurysms [1].

### 1.1. Saccular Aneurysms

The shape of aortic aneurysms is generally classified as being either fusiform or saccular. Saccular aneurysms are further divided into concentric and eccentric types. Fusiform and concentric saccular aneurysms result from a generalized weakening of the entire circumference of the affected vessel. In contrast, eccentric saccular aneurysms arise from a focal weakness due to an intrinsic abnormality or an extrinsic process that causes localized arterial damage. While fusiform abdominal aortic aneurysms (AAAs) frequently result from arterial wall degeneration, saccular aneurysms of the abdominal aorta (sa-AA) appear to have a more varied aetiology. This includes trauma, aortic infection, inflammatory diseases, degeneration of a penetrating atherosclerotic ulcer, and previous aortic surgery [2,3]. Most infective native aortic aneurysms (INAAs), including numerous strains of bacteria and fungi (e.g., *Candida*, *Aspergillus*) as causative agents [3], exhibit an eccentric saccular shape, as the latest 2024 Delphi consensus has deeply analyzed [4]. Fusiform or concentric saccular aneurysmal morphologies are, on the other hand, predominantly linked to atherosclerotic degeneration of the entire vessel wall [3]. Eccentric saccular morphology is characteristic of renal and intracranial (cerebral) aneurysms [5] but is less frequently observed in non-cerebral locations, except the renal arteries [6].

Overall, sa-AAs are relatively rare, mostly reported in case reports and small case series regarding their clinical presentation and aetiology [7]. As Karthaus et al. [8] described in their review, reported incidences of sa-AA may vary from 1.5% to 5.0% in the general population. The majority of saccular aneurysms in this cohort were located in the descending thoracic aorta, with only 24.2% in the abdominal aorta. While case series have suggested a varied aetiology for sa-AA, Shang et al. [9] found that the majority (81.1%) of these pathologies were caused by atherosclerosis (degeneration). Only 3.7% were due to trauma, 1.2% were caused by infection, 1.0% by arteritis, and 13.1% had an unclear aetiology.

### 1.2. Penetrating Aortic Ulcer (PAU)

Among the various types of focal non-infected lesions affecting the abdominal aorta, the penetrating aortic ulcer (PAU) is a well-documented example that offers insight into the potential behaviour and risk associated with localized aortic wall disruption [10].

Initially described by Shennan [11] in 1934, a penetrating aortic ulcer (PAU) was further characterized by Stanson et al. [12] in 1986, marking its recognition as a distinct aortic pathology. A PAU is identified as a focal ulcerative lesion occurring in an atheromatous plaque through the elastic fibres of the media lamina and extending deeply into the intima. Subsequently, this process can lead to localized pathology as aortic dissection (AD) and varying degrees of intramural hematoma (IMH) formation within the aortic wall, with an incidence of approximately 2–3% [13,14,15,16]. However, recent studies indicate that PAUs can progress into pseudoaneurysms and saccular aneurysms, characterized by adventitial expansion beyond the initial confines of the aortic wall. In fact, during the long-term course of PAUs, local weakening of the aortic media due to the penetrating ulcer may result in aortic dilatation. Previous studies reported that up to 50% of patients developed significant aortic aneurysms during follow-up [17]. While classically described in the descending thoracic aorta, PAUs have also been reported in the abdominal segment, albeit less frequently. These lesions may remain stable or progress to pseudoaneurysm or saccular aneurysm formation, with varying clinical consequences. Recent studies have hypothesized that some saccular aneurysms, particularly those associated with atherosclerotic disease, may originate from PAUs [18,19].

A recent 2019 literature review [20] identified 298 cases of abdominal PAUs between 1974 and 2015, noting that the affected patients were typically elderly males with multiple cardiovascular risk factors [2,20,21,22,23,24,25]. Also, they generally have multiple serious comorbidities such as concomitant coronary or peripheral arterial disease (30.7%) [20] and quite high smoking incidence [23,24,25,26]. This data confirmed the 2005 review of the literature by Batt et al., which deeply described the PAUs of the DTA and the AAs [19].

While thoracic PAUs are often symptomatic, causing pain (52%) or acute lower limb ischemia because of distal embolism (12%) [27], those in the abdominal aorta are frequently asymptomatic and discovered incidentally [21]. Lower limb embolism complicating PAUs of AAs was quite common. When there are no signs suggesting a cardiac origin, the discovery of a lower limb embolism should prompt a search for an aortic cause, and in particular, PAUs. Ruptures associated with PAUs are also variable [19,22]. Nonetheless, the risk of rupture, although lower than that for thoracic PAUs, remains clinically significant and underscores the importance of appropriate surveillance.

Nevertheless, the Mayo Clinic Classification [14] identified the three radiological diagnostic criteria for PAUs on contrast-enhanced CT scans: transmural rupture with extra-aortic hematoma; well-defined ulcer crater in the aortic wall; and subadventitial saccular aneurysm extending beyond the wall of the aorta [12].

Therefore, the propensity of PAUs toward aneurysm or pseudoaneurysm development must be carefully monitored during long-term follow-up, even in asymptomatic patients [11].

The management of uncomplicated penetrating aortic ulcers (PAUs) remains a subject of ongoing debate due to limited long-term outcome data and heterogeneous clinical presentations. In the 2024 ESC guidelines, treatment decisions are increasingly guided by morphological high-risk features, including ulcer depth, diameter, and associated intramural hematoma (IMH). However, despite these recommendations, substantial evidence gaps persist regarding the natural history and prognostic trajectory of PAUs and IMHs, particularly in asymptomatic patients. To contextualize this uncertainty, insights can be drawn from major randomized trials in abdominal aortic aneurysm (AAA) management—namely EVAR I and II—which demonstrated no survival benefit for early intervention in low-risk patients. These findings reinforce the rationale for a cautious, individualized approach in PAU treatment. Accordingly, a rigorous risk–benefit assessment is essential, and watchful waiting may represent a viable strategy in selected cases with stable imaging and no clinical symptoms. Collectively, these considerations contribute to a more nuanced and pragmatic framework for decision-making in the absence of robust prospective data.

## 2. Indications for Treatment and Synthesis of the Available Literature (Open Surgery vs. Endovascular Therapy)

The management of focal non-infected pathologies of the abdominal aorta remains a subject of debate. Unlike fusiform abdominal aortic aneurysms, the inconsistent definitions and limited information regarding the natural history of sa-AAA have prevented the establishment of universally accepted practice guidelines for their management. As emphasized in the latest 2024 European Society of Vascular Surgery (ESVS) guidelines [27], the focal nature of these diseases makes them ideal candidates for endovascular repair (class of evidence IIa—level C). Moreover, the Society for Vascular Surgery (SVS) [28] referred to aneurysm diameter as an indication for treatment, suggesting using a smaller diameter compared to fusiform aneurysms. Consequently, the management of saccular aneurysms is likely heterogeneous amongst different centres and different operators.

The main aspect surrounding the natural sa-AA is their well-documented rupture risk of saccular aneurysms. Recent publications, including the SVS and ESVS guidelines [27,28], highlight this as a primary concern for sa-AAA repair, regardless of aneurysm size. In a related study, Vorp et al. [29] used computer-generated models to demonstrate that asymmetry is as critical as diameter in determining aortic wall stress and rupture risk. Therefore, size-based criteria for AAA repair are less applicable to saccular AAAs [30]. As Pantoja et al. [31] brilliantly summarized, the rupture mechanism is influenced by low wall shear stress, which enhances the atherogenicity of the intimal layer, promotes abnormal vessel remodeling, and facilitates intraluminal thrombus formation. These mechanical–cellular interactions, in turn, increase metalloprotease activity, thereby reducing the strength of the vessel wall and promoting the risk of rupture. Due to the uncertainty surrounding a potentially elevated rupture risk, early treatment may be considered, with a lower diameter threshold for elective repair compared to standard fusiform AAAs [27,28,32,33].

However, the behaviour of these focal non-infected lesions of the abdominal aorta remains a critical aspect requiring further study to fully understand the impact of size on their progression and prognosis. Previous studies have suggested that for patients characterized by advanced age, major comorbidities, and increased atherosclerotic burden, open aortic surgery may not be the optimal treatment option [34,35]. Additionally, even though sa-AAAs usually do not display degenerative atherosclerotic process or laminar thrombus that may interfere with device attachment to the aortic wall, making it an ideal candidate for endovascular repair, there is no consistent comparative research excluding isolated case reports [36,37,38] that could support the choice of endovascular aneurism repair (EVAR) instead of open surgery repair (OSR). Furthermore, as we will explore in the next sections, anatomical hurdles may nonetheless exist and should be carefully taken into consideration. Also, data on this rare aneurysmal defect are scant, and the appropriate timing and method of treatment are not well defined; also, long-term results are not yet available. Our current understanding of the natural history of these conditions is, therefore, limited, and treatment selection is still based on incomplete information. However, in a recent multicentric contemporary experience reported by Lomazzi et al. [32], the authors showed durable positive outcomes, with no EVAR failures and no aneurysm-related mortality beyond the early postoperative stage, indicating the effective durability of EVAR for saccular lesions of the abdominal aorta (sl-AAs).

Otherwise, the optimal therapeutic regimen for PAU disease is controversial. While asymptomatic cases may be managed conservatively with close follow-up, early intervention is recommended when the PAU is complicated with aneurysm expansion, rupture, embolic symptoms, or uncontrolled pain. Indeed, the course of PAUs of AA disease is variable and it is mostly influenced by the risk of rupture. The evaluation of the risk of rupture is deeply supported by Vorp et al. [29], who reported the importance of the shape and symmetry, in addition to the size of the abdominal PAUs. The usual description of computed tomography angiography (CTA) includes the measurement of the maximum aortic diameter at the ulcer site, the depth of the ulcer, and the length of the intimal defect (width) at the ulcer site. Some features could be suggested as complicated, including a growth rate in abdominal PAUs of about 3 mm/y [39,40], a coexisting extra-aortic hematoma which measures > 20 mm in width or >10 mm in depth or which progresses into a total abdominal aortic diameter of 50mm or more [23,39,40]. Ganaha et al. [41] also reported that the size and depth of PAUs are predictors of disease progression.

Despite these efforts, there is still a lack of predictors of the natural history and behaviour of PAUs, and no standard treatment has been established. During the past few years, PAUs have increasingly been treated more aggressively, preferentially surgically. In 1992, Hollier et al. [42] recommended surgical intervention for PAUs of AAs, irrespective of their size, considering the risk of rupture. In addition, a small case series regarding PAU endovascular treatment is rapidly increasing. In 2005, Ford and Farber et al. [43] documented 133 patients treated endovascularly, and unsuccessful deployment of the stent graft was reported in only three patients, resulting in a procedural mortality rate of 3–4%; three patients developed spinal ischemia, and three experienced a stroke. Also, in the recent review of the literature by Kotsis et al. [20], most authors reported endovascular stent graft repair as the treatment of choice of PAUs of AAs (70%), while open surgical repair was performed in 30% of patients, even if it is associated with higher operative morbidity and mortality due to the patient’s poor general condition and comorbidities.

## 3. Challenges with Endovascular Repair of Focal Lesions of the Abdominal Aorta

Although a prospective, randomized long-term comparison of open versus endovascular AAA repair for such focal pathologies has not yet been conducted, the endovascular approach has demonstrated advantages in terms of morbidity [44,45]. For these reasons, endovascular stent-graft placement has emerged as a promising, less invasive alternative to open surgery in the treatment of aortic focal non-infected lesions of the abdominal aorta, including sa-AAAs and PAUs. The use of different endovascular techniques undoubtedly has been increasing in the last few years [46], as Table 1 shows.

Historically, endovascular repair of fusiform AAAs using aorto–aortic stent grafts has faced criticism due to a high incidence of migration and/or secondary type I endoleaks [51,52]. However, the application of tubular devices has since been reconsidered for well-selected patients with focal aortic pathologies: indeed, saccular abdominal aortic aneurysms may often lack a degenerative atherosclerotic component and laminar thrombus, which in turn may facilitate optimal device attachment to the aortic wall, making it a prime candidate for tube graft repair. Furthermore, PAUs and sa-AAAs are focal lesions, limited to a short aortic segment with typically small, eccentrically placed necks in the aneurysm, which makes them particularly amenable as “ideal” targets that can be completely excluded with a single-piece stent graft. While a bifurcated endograft might treat a saccular AAA, there is a risk of limb compression if the distal aorta is non-aneurysmal and the aortic bifurcation is narrow.

For these reasons, it is essential to consider various anatomical and physical factors when selecting the appropriate endovascular graft. Currently, the availability of a range of stent-graft sizes in both length and diameter facilitates the accommodation of diverse aortic anatomies. The implementation of the double-tube technique and/or the addition of an aortic cuff provides each tube graft with proximal and distal contact zones, enabling potential telescope-like movement between the devices if the attachment at both necks is secure. This increased structural rigidity may reduce the risk of migration. Differences in stent graft diameter of up to 12 mm can be accommodated by deploying a larger stent graft within a smaller one [46]. Additionally, inaccuracies in preoperative length measurements can be addressed by adjusting the overlap between the tubes using telescoping techniques [38]. Accurate proximal and distal deployment of cuffs, measuring 50 mm and 70 mm in length, respectively, can achieve a total length between 100 and 110 mm, effectively covering the distance between the renal arteries and the aortic bifurcation [37]. This configuration ensures sufficient overlap, thereby minimizing the risk of type III endoleak.

Furthermore, another persuasive issue for the endovascular approach in focal abdominal aortic repair is the use of custom-made devices in challenging cases such as infrarenal lesions [38,46]. These devices increasingly ensure optimal treatment and precise, patient-centred preoperative studies, even though they force the laborious and time-consuming procedures required for their preparation and deployment [49].

In 1995, Professor Wolf Stelter [46] developed the multimodular composite concept to position a bifurcated stent graft into the aortic bifurcation, extending it proximally with an aortic cuff. This composite system was the impetus for the concept of “double tube” stent grafts, which nowadays are used in the sa-AAA repair [46]. Typically, this involves placing a distal tube graft exactly into the aortic bifurcation and a proximal tube graft directly beneath the renal arteries.

Moreover, Mazzaccaro et al. [53] reviewed 53 patients who were treated with various devices (Endologix, Vanguard, and Talent). Their results suggested that reintervention and endoleaks were more frequent in the group of patients receiving a single endograft.

In 2007, Ruppert et al. [47] first introduced the term “trombone” technique for 45 patients treated with double-tube stent grafts. The overlap of the two stent grafts was calculated to be 4 to 8 cm. In the early phase, custom-made Zenith stent grafts (Cook Europe, Bjaeverskov, Denmark) were used; later on, no longer commercially available Powerlink stent grafts (Endologix Inc., Irvine, CA, USA) were employed. Their medium 20-month follow-up showed 9.8% (4/41 patients) of endoleaks, one distal type I, two type II, and one type III. They concluded that a favourable outcome with this technique can be met with proper patient selection and aortic anatomic criteria.

This technique was further endorsed by Jones et al. [48], who reported excellent early outcomes in eight patients treated with Zenith tube devices. The authors concluded that the trombone technique is less invasive and allows for better length adjustment with fewer flow issues in small-diameter distal aortas.

Later, York et al. [37] proposed a monocentric retrospective observational study of 147 patients in which AneuRx aortic cuffs (no longer commercially available) were used in a “stacked” configuration to effectively treat sa-AAA. A key benefit of the “stacked” technique is the ability to tailor the endograft length to the patient’s anatomy, proving effectiveness for this rare aneurysm type. To prevent a type III endoleak at the overlapping cuff junctions, it is crucial to overlap the devices by at least 1.5 to 2.0 cm. No endoleaks were observed during the 12-month follow-up using this method.

In 2016, the report of Giagtzidis et al. [38] sought to retrospectively review the experience in using the aortic extension cuffs of the Endurant (Medtronic AVE, Santa Rosa, CA, USA) endovascular stent-graft device in sa-AAA repair. A single 70 mm long Endurant aortic extension was deployed in five cases of sa-AAAs, and aortic cuffs were used with the “telescopic” (double tube) technique. One type II endoleak was encountered during the follow-up period (around 20 months). The authors believed that the technical characteristics of the Endurant cuff increase the stiffness of telescopic construction, reducing the likelihood of migration.

The advent of custom-made (CM) technology in sa-AAA repair was recently investigated by D’Oria et al. [49] in 2019, describing elective endovascular repair in patients with narrow aortic bifurcation using CM unibody conical endografts (JOTEC Extra Design Engineering stent graft—JOTEC GmbH, Hechingen, Germany). All the devices had been constructed by referring to preoperative CTA to achieve a 25% oversizing of the device as compared to the native aortic diameter at the level of the intended necks, while the total device length was 5 mm longer than the “lower renal-to-terminal aorta” distance. The technical success rate was 100%, with freedom from any endoleak or reinterventions or conversions during their longest follow-up period of 12 months.

In conclusion, the use of standard bifurcated EVAR platforms should still be seen as the first-line option for endovascular repair of focal disease of the abdominal aorta when technically and anatomically feasible. However, it becomes evident that patients with focal aortic pathology may be unsuitable for the use of a bifurcated device for various reasons, especially because the limbs of the device might be prone to compression, kinking, and thrombosis in an otherwise narrow distal aorta (which is often seen in these cases). For these authors, the key to a successful outcome of focal non-infected lesions of the abdominal aorta is appropriate patient selection, choosing the most suitable device and procedure for the patient.

## 4. Case Examples and Technical Solutions

In the following paragraphs, we provide a brief overview of actual cases encompassing a broad range of technical solutions, ranging from standard EVAR to complex fenestrated-branched solutions. The objective is to present the readers with some of the technical issues that may be encountered and how to solve them.

### 4.1. Infrarenal EVAR

As already underlined, the EVAR procedure is rapidly gaining broader acceptance for the management of saccular abdominal aortic aneurysms and penetrating aortic ulcers of the abdominal aorta. Despite many different techniques that have been reported in recent years, yielding remarkable results, there is still a lack of large prospective studies that analyze the differences and outcomes between open and endovascular treatments, which would provide a robust scientific foundation to support the liberal adoption of endovascular treatment. Furthermore, the decision to opt for endovascular repair should take into consideration potential anatomical hurdles as well as the patient’s fitness for surgical repair and their life expectancy. Based on the available data, however, standard EVAR certainly has proved to yield satisfactory outcomes at least at mid-term follow-up.

In the standard EVAR scenario, some authors in [54] highlighted the AFX device as being well suited for saccular AAA cases with a narrow aortic bifurcation, where conventional bifurcated endografts’ iliac limbs might compete within the constricted lumen, thereby making the repair potentially prone to stenosis or thrombosis. A thrombotic or irregular infrarenal neck (such as reverse tapered or bulging neck) can also be effectively handled by AFX’s functioning mechanism, accommodating the irregularities without applying excessive radial force. However, previous studies [55,56] also showed a high incidence of type III endoleaks, to such an extent that the US Food and Drug Administration issued a class I voluntary recall of AFX systems in 2018. In contrast, recent research has indicated excellent outcomes with AFX, showing a low incidence of type III endoleaks [57]. Furthermore, after AFX2 was introduced in February 2017 in Japan, more than three years of outcomes data have been collected by Hoshina et al. [50] in Japan with 1807 AFX2 cases, of which 48 cases (27%) involved saccular aneurysms. One of the main results was, however, the migration risk associated with large AAA diameters, which occurred in five cases of aortic cuff and main body migrations. Four cases showed this type of migration, named sideways displacement, 3 years after EVAR. The authors suggested two hypotheses: sideways deformation that may occur within a sufficiently large AAA sac, and the billowing fabric of the aortic cuff that may create a reverse windsock effect, increasing the distraction forces between the main body and the aortic cuff. They reported a type IIIa endoleak in a patient with a notably large terminal aorta (28 mm in diameter). The authors theorized that the ample space around the terminal aorta allowed the main body to billow downward, causing the endograft components to decouple. This scenario illustrates why AFX may be particularly suitable for saccular AAA with large aneurysm risks [58].

Since 1998, endovascular criteria for EVAR procedures have been extensively researched, establishing essential anatomical considerations for aneurysms and surrounding vessels that are crucial for planning complex aortic procedures. Key measurements from imaging studies include the lengths of the proximal and distal necks, the distances between the renal arteries and the aortic/iliac bifurcations, and the diameters and configurations of the necks. Other important factors assessed include the dimensions of the common and external iliac arteries, the angulation of the proximal neck, vessel tortuosity, and the presence of calcification and mural thrombus [59].

**Figure 1:**
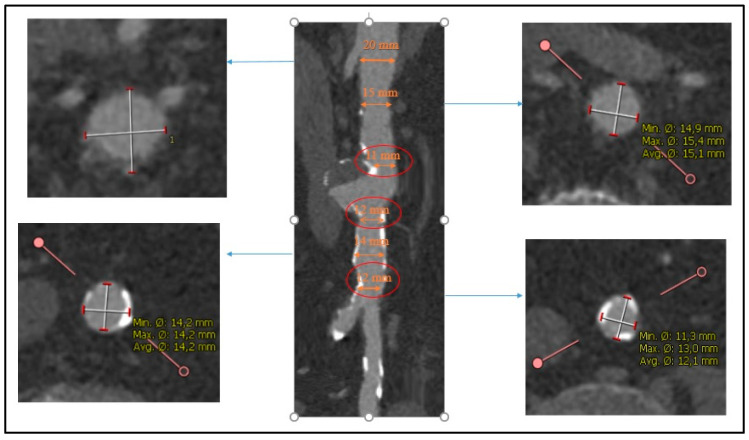
Preoperative computed tomography angiography (CTA) showing saccular aneurysm of the abdominal infrarenal aorta with very narrow and highly calcified aortic bifurcation.

**Figure 2:**
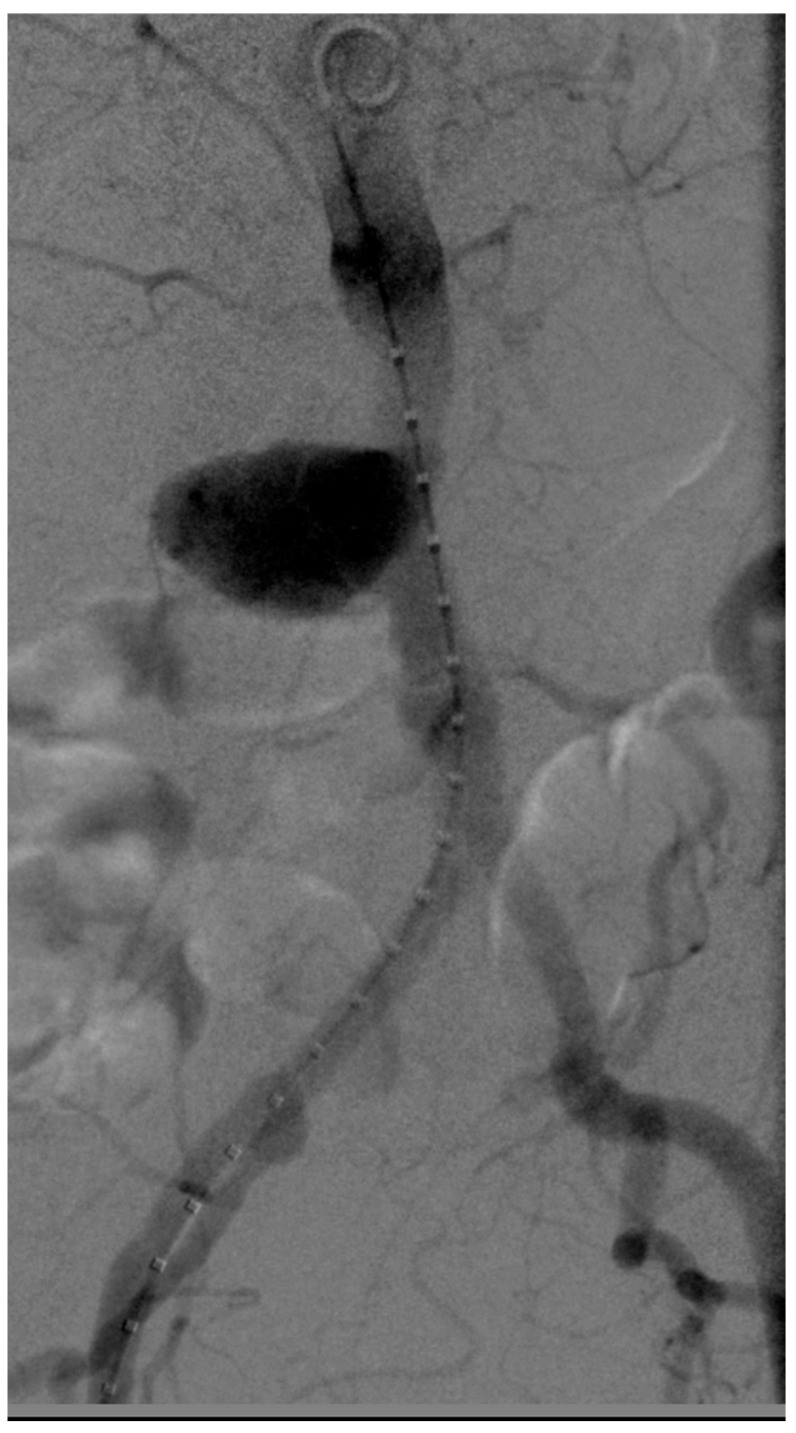
Same case. Intraoperative angiography confirms the location of the focal lesion in the infrarenal abdominal aorta.

**Figure 3:**
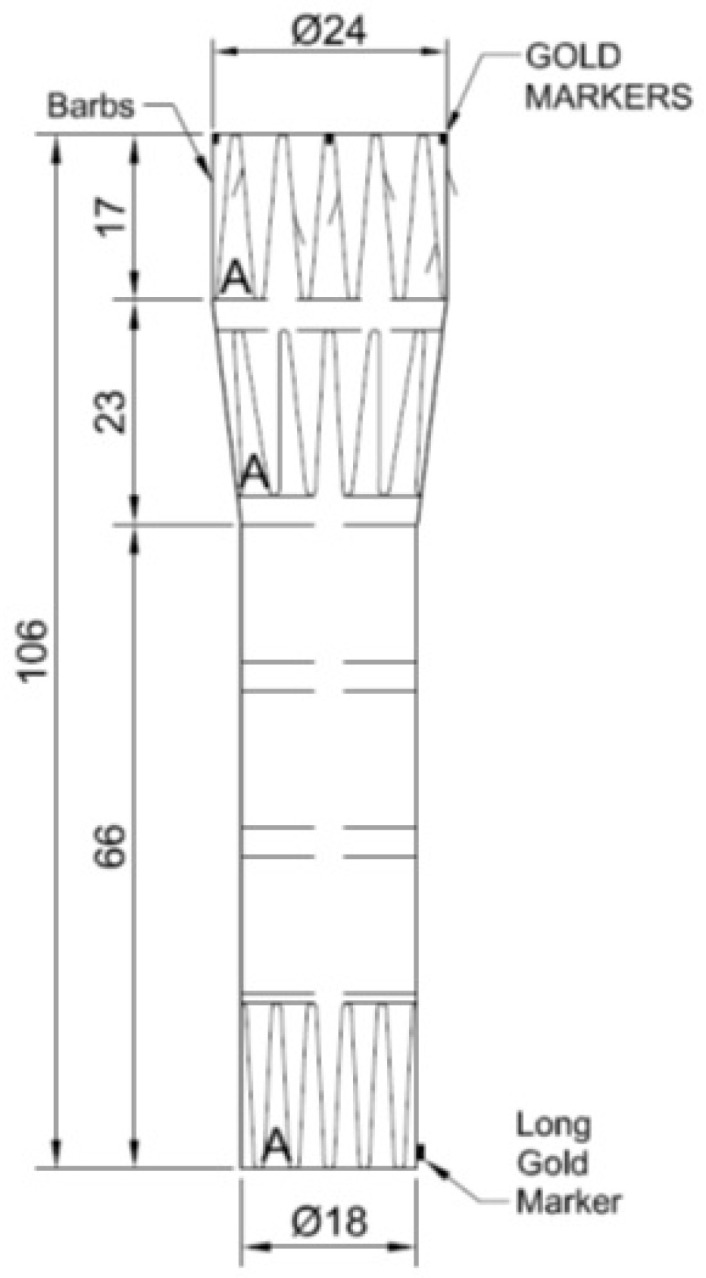
Same case. Technical drawing of the custom-made stent graft with unibody conical configuration. (The letter A indicates sealing stents with high radial force).

**Figure 4:**
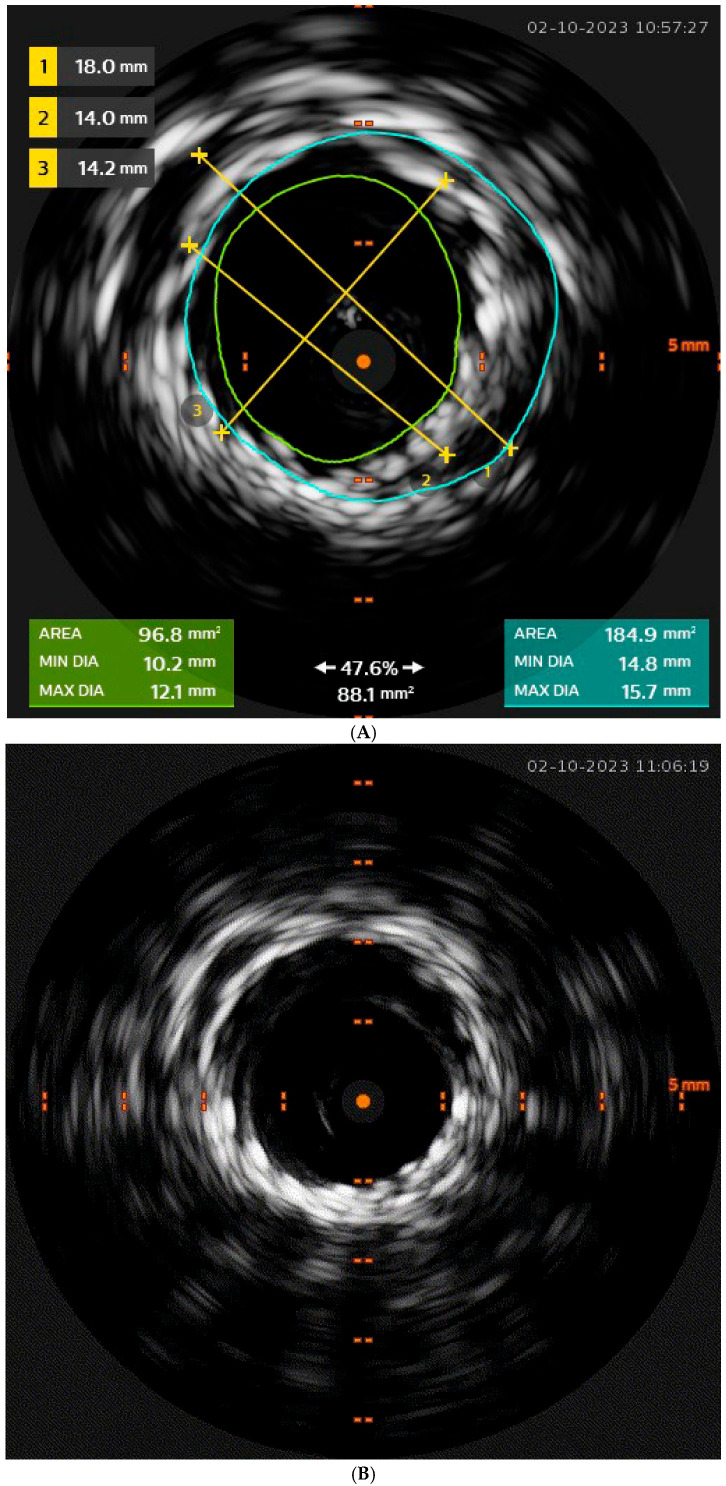
(**A**) Same case. Intraoperative IVUs showing the incomplete opening of the stent graft and its diameter in line 2 at the distal edge (light blue circle), with non-circumferential apposition to the inner aortic wall as line 1 diameter. The green circle represents the residual inner aorta lumen. (**B**) Same case. Intraoperative IVUS after molding of the distal edge of the stent graft with a compliant aortic balloon showing full circumferential apposition to the inner aortic wall.

**Figure 5:**
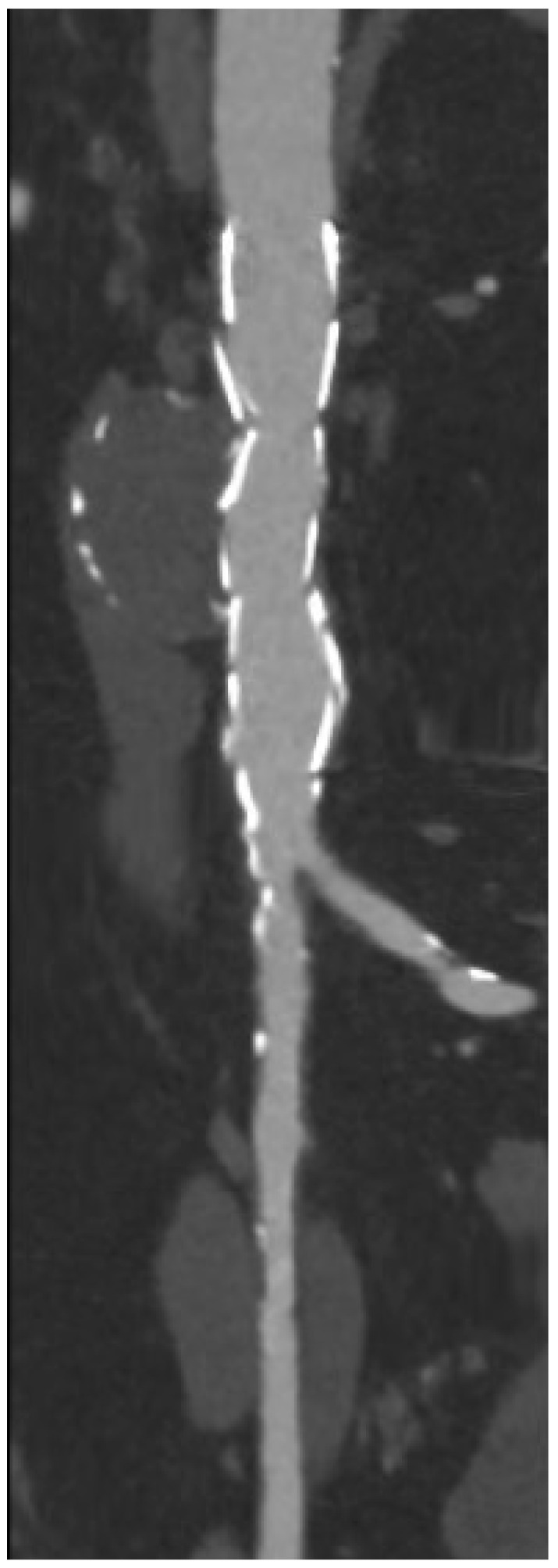
Same case. Follow-up CTA showing complete exclusion of the saccular aneurysm, optimal apposition of the endograft to the aortic walls, and preservation of both iliac axes.

Notwithstanding the possible existence of anatomical challenges, as delineated in the previous paragraphs, when the indication for repair is made and the planning is suitable, there are different technical approaches available. In the absence of data, each of the techniques and materials mentioned in the previous paragraphs may be considered as valid options in the appropriate scenario. The selection of materials and techniques should be carefully considered and evaluated based on the centre’s experience and the confidence of its surgeons [38,45,47,49,53]. Lastly, a recent review in the literature on PAU endovascular treatment and outcomes [60] revealed an innovative alternative procedure using the implantation of balloon-expandable covered stents (BeGraft, Bentley InnoMed GmbH, Hechingen, Germany) [61,62]. This approach was primarily guided by anatomical assessment and surgeons’ preferences, as well as the presence and severity of iliac occlusive disease. The use of balloon-expandable covered stents may provide the benefit of a lower profile design, avoiding unnecessary treatment of extensive aortic segments. Favourable mid-term outcomes regarding technical success and patency have been validated; however, this approach may not be currently endorsed as first-line pending further validation. As discussed before, the use of CM endografts with a reverse conical configuration may sometimes be a feasible technical option to avoid potential risks of flow competition in the iliac limbs. However, in cases with extremely narrow and calcified aortic bifurcations, as often seen in focal abdominal aortic pathologies (Figure 1 and Figure 2), the stent graft may infold even after pre-emptive kissing balloon angioplasty (Figure 3). Such infolding may be predisposed to a type 1B endoleak, and additional imaging techniques may be used to understand the endograft behaviour and guide further intraoperative manoeuvers [63]. For instance, IVUS may help demonstrate the areas of inadequate graft-wall apposition and guide further expansion of the stent graft (Figure 4A,B and Figure 5).

### 4.2. Fenestrated-Branched Options for Disease Involving the Para-Visceral Aorta

**Figure 6:**
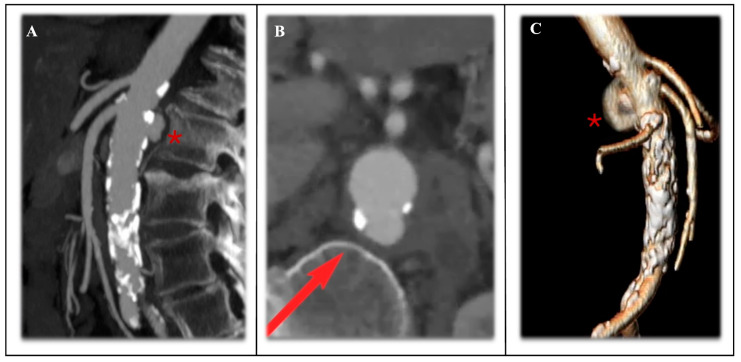
Preoperative computed tomography angiography (CTA) of the para-visceral penetrating aortic ulcer (PAU) positioned at the level of the superior mesenteric artery (SMA) in MPR projection ((**A**) red asteriks), in planar 2D vision ((**B**) red arrow, in the posterior portion of the aorta) and in the 3D volume rendering recontruction ((**C**), red asterisk).

The para-visceral aorta represents one of the most challenging regions for endovascular repair. Focal disease here is a rare entity combined with complicated penetrating aortic ulcers (PAUs) and saccular aneurysms (Figure 6), while descending thoracic or infrarenal abdominal aorta diseases often directly involve this specific segment or require a landing zone at this level [64,65].

Robust data are scarce, consisting largely of single-centre reports and small retrospective series [64,65,66]. Open surgical repair by thoraco-phreno-laparotomy with supra-celiac clamping was once the only definitive therapy, yet it carries notable pulmonary, renal and spinal cord morbidity [67]. These challenges have favoured endovascular strategies consisting mainly of fenestrated and branched endovascular aortic repair (F/B EVAR), already regarded as first-line treatment for anatomically suitable complex abdominal and thoracoabdominal aneurysms (cAAA and TAAA) in high-risk patients as reported by the latest European Society for Vascular Surgery guidelines [27]. Although large dedicated series are slacking, single-centre experiences demonstrate high technical success and encouraging early to midterm durability with custom-made fenestrated devices [65,68,69] off-the-shelf branched grafts [70,71] and for mainly urgent cases, physician-modified/in situ laser fenestrations [72,73,74,75] and parallel graft configurations. The present chapter synthesizes that evidence and provides a pragmatic framework for indication, planning, graft selection and technical execution.

Indications for repair should be based not only on maximum diameter and morphology of the aorta, but also on the presence of symptoms/rupture. In the case of asymptomatic lesions without signs of rupture, they may reasonably await the manufacture of a patient-specific fenestrated graft, mainly due to the presence of a small aortic diameter at this level with reduced gap distance [76], favouring fenestrated repair over directional branches. Notably, waiting times should be reasonable with known possible complications/ruptures during the interval time between manufacturing and implant [77,78]. As well as in the infrarenal segment [79,80], penetrating aortic ulcers are often associated with a healthy calcified aorta and small/diseased iliac accesses [81,82] and adjunctive devices such as preloaded grafts might be considered in order to reduce iliac sheath size and occlusion time. Once any rupture signs [83] appear, such as back or abdominal pain, periaortic haematoma or rapid sac expansion, the repair should not be deferred. Urgent lesions of this sort can be frequently treated with off-the-shelf multibranch devices with some anatomical constraints that might be kept into consideration, as well as newer devices such as low-profile multibranched endografts and partial deployment techniques [84,85] might be useful to overcome these issues. A smaller but crucial subset in emergent cases where there is no possibility of off-the-shelf repair, physician-modified/in situ laser fenestrations and parallel graft reconstruction, usually in chimney or periscope configuration, remain lifesaving.

**Figure 7:**
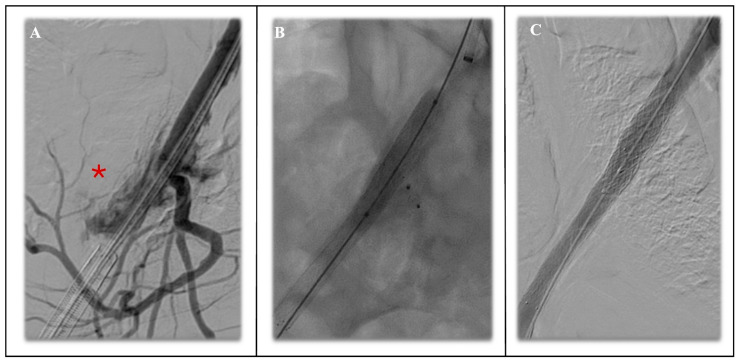
Evidence of iliac rupture during the advancement of large bore custom-made fenestrated endograft (**A**) with rupture at the level of the right external iliac artery (red asterisk); solved by endovascular repair with covered self-expanding covered stent in external iliac artery, plug embolization of the interna iliac artery and balloon-expanding covered stent in common iliac artery (**B**), confirmed by the angiogram with successful exclusion of the rupture (**C**).

Pre-operative planning remains the pivotal stage in these repairs, on this slice-millimetre computed tomography angiography reconstructed along the centre line of flow. The first anatomical aspect is the proximal landing zone, which must be long and healthy enough to accept a durable seal; a straight segment of at least 20 mm above the coeliac origin is considered the lower limit for fenestrated repair [86]. The second aspect should be the origin of visceral vessels and the diameter of the aorta at this region: in the case of a narrow aorta favouring wall apposition, fenestrations can be aligned safely; otherwise, directional branches provide a more favourable configuration. In these latter cases, attention should be paid to vessel orientation and to avoid covering as much of the healthy aorta as possible [87,88,89], together with segmental arteries and an augmented risk of spinal injuries. Distal landing options must be examined. A calcified or narrowed distal aorta compromises bifurcated limbs; in such cases, a single fenestrated customized tube graft can be planned with an inverted-limb bifurcated graft in the case of a short-segment aorta. Finally, access vessels set practical limits: contemporary branched systems usually demand 22–24 French (F) delivery profiles; iliac or axillary arteries with an internal diameter below seven millimetres may carry risk for dissection or rupture (Figure 7). When the iliac access is hostile, a low profile fenestrated custom-made device should be planned, and additional techniques such as paving and cracking [90] or iliac conduit can be considered [91,92].

**Figure 8:**
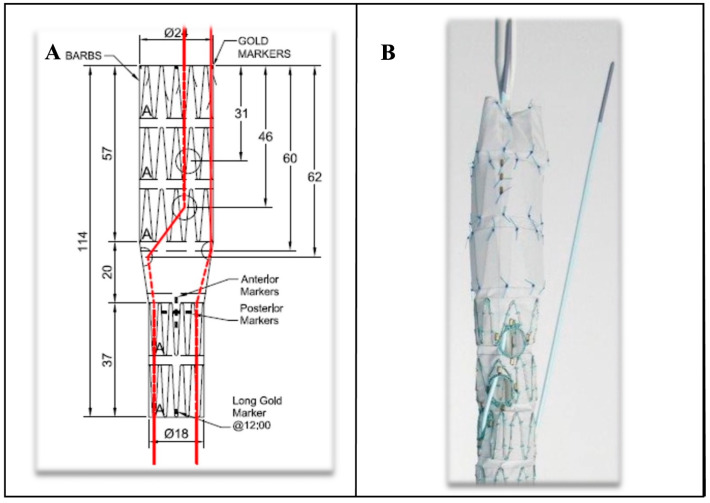
Custom-made-device fenestrated endograft planning (**A**) provided by Cook Medical (Cook Medical, Bjaeverskov, Denmark) with 4 fenestration endografts with pre-loaded catheter for left renal fenestration and for right renal (from below) and superior mesenteric artery (from above); the endograft is shown as in panel (**B**) and loaded into a 22 F sheath. (The letter A indicates sealing stents with high radial force. The red lines indicate the pre-loaded catheters).

The customization process has been refined to face anatomically specific requirements. Pre-loaded guidewire [69] systems now allow for threading 0.018-inch nitinol guidewires through each fenestration (Figure 8), emerging at the delivery system hub. During the operation, a 6F introducer sheath snares the preloaded wire outside each fenestration and is then used to parallel canulate each targeted visceral vessel (TVV).

Bridging stent technology has also matured. Balloon expandable covered stents provide high radial force and precise length control and newer devices with 6F compatibility enhance the usage of low-profile delivering systems; self-expanding PTFE-lined stents offer compliance in sharply angulated vessels. A hybrid stent-graft technique, where a balloon expands proximally into the graft and self-expands distally, can merge precision with flexibility, especially for a branched configuration [93].

**Figure 9:**
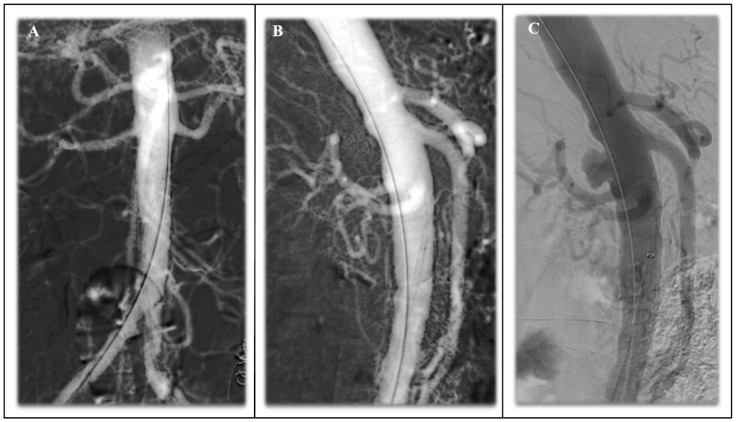
Anterior–posterior view (**A**) and lateral–lateral view (**B**) of pre-implanting of the endograft using carbon dioxide double subtraction angiography. Latero–lateral view using iodinated contrast media (**C**) to show the para-visceral PAU in the posterior wall of the aorta.

**Figure 10:**
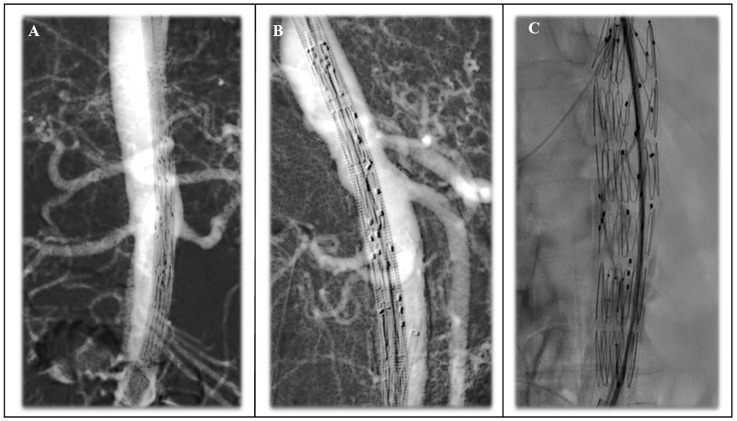
Precise positioning of the endograft according to TVVs ostia and marker of the fenestrated graft positioning according to anterior–posterior view (**A**) for renal arteries and latero–lateral view for superior mesenteric and celiac trunk (**B**) and complete deployment of the endograft (**C**).

Under general anaesthesia, the groins are accessed percutaneously or by surgical access and pre-closed with suture-mediated devices. In case of preloaded wire, branchial surgical access can be performed. Systemic heparin is given to achieve an activated clotting time above 200 s. A stiff, pre-curved Lunderquist guidewire is parked just above the aortic valve. The fenestrated main body is advanced under fluoroscopy that is synchronized with the pre-operative computed tomography angiography (CTA) fusion imaging and positioned after performing anterior–posterior and latero–lateral views (Figure 9). The endograft is positioned when the markers overlay the projected visceral ostia. After partial release, alignment is scrutinized in antero–posterior and oblique views (Figure 10). With pre-loaded wires or hydrophilic catheters, the renal arteries are sequentially cannulated while the superior mesenteric arteries and celiac trunk are cannulated using the above access. Balloon expandable covered stents are inserted but left undeployed until all vessels are secured. Full release of the main body follows, then each bridging stent is deployed, flared, and post-dilated. A low-pressure aortic cuff balloon ensures uniform fabric apposition to the aortic wall and, given the non-pathological nature of the infrarenal aorta, a tube-fenestrated graft is enough in many cases to ensure technical success and PAU exclusion. Cone beam computed tomography or post-operative CTA to confirm the absence of an endoleak and kinks in the materials, together with satisfactory bridging stent-graft flaring.

When thoracic coverage spans well above the celiac trunk, protocol for spinal cord injuries should be attentively considered, such as staging, cerebrospinal fluid drainage, high mean blood pressure and attention to preserve collateral circulation through left subclavian and hypogastric arteries [89,94,95,96].

Branched repair with off-the-shelf multibranched devices mirrors the procedure seen for fenestrated repair but adds the complexity of antegrade TVVs cannulations. Many teams now expose only the proximal third of the multibranch body, stent the coeliac and SMA antegrade, then advance and expose the renal branches for sequential cannulation, a partial approach that consistently keeps short visceral ischaemia and provides efficacy in deploying these grafts into an aortic lumen smaller than the 25 mm cut-off [84,85,87]; however, may exposs the patients to higher rates of postoperative renal vessel thrombosis [97,98].

In selected cases, before the main body is introduced, a five-French catheter/introduced sheath is advanced from the contralateral femoral artery into the cavity of the saccular aneurysm/PAU. After deployment of the main endograft before distal ballooning, the 5F introducer sheath/catheter becomes the channel for coil packing the aortic lesion [66,80,99]. Completion angiography must show perfect exclusion and unimpeded branch flow.

## 5. Conclusions: Evidence Summary, Unmet Needs and Future Directions

The management of focal non-infected pathologies of the abdominal aorta still lacks consensus. Endovascular repair using tube stent grafts offers benefits like reduced recovery times but carries risks of migration and endoleak due to graft rigidity. These complications can influence long-term success. In this context, the use of endovascular bifurcated grafts may provide a more effective solution for treating these focal aortic pathologies. It is essential to achieve optimal sealing regions through anatomical studies of aortic morphology. Additionally, understanding the anatomical characteristics of focal lesions in challenging necks or para-visceral locations is indeed crucial in device choice. Off-the-shelf devices are favoured for their time and cost efficiency, but new endovascular technologies like FEVAR and custom-made devices enhance treatment success and patient safety. These innovations provide stent grafts in various lengths and diameters, accommodating different aortic anatomies and reducing the risk of type III endoleaks. Although complicated PAUs and focal saccular aneurysms rarely arise in the para-visceral aorta, the consequences of rupture in this segment might be extremely severe. Experience borrowed from complex abdominal and thoracoabdominal aneurysm repair demonstrates that fenestrated and branched devices can be deployed safely when anatomical criteria are respected. Elective patients derive the greatest benefit from a fenestrated graft, while urgent cases can be treated confidently with off-the-shelf multibranch systems, reserving other types of repairs for emergent or bail-out cases. While early outcomes of these interventions are promising, it is crucial to acknowledge that limited aortic coverage can still impede effective symptom relief and lead to complications such as aneurysm expansion or rupture. Therefore, further long-term studies are essential to consolidate the technical results and evaluate the durability of various graft options.

## Figures and Tables

**Table 1:** Summary of studies on endovascular treatment of saccular AAA.

Study (First Author)	Years	Number of Patients	Primary Pathology	Endovascular Technique	Device Used	Follow-Up (Months)	Mortality	Postoperative Complication
Stelter [41]	1995	201	AAA	“Double Tube” technique	Stentor/Vanguard device (178) Talent endograft (23)	24	3.5% aortic-related: MOF (5) Hemorragic complications (2)	9% Endoleak: 9/201 proximal 16/201 distal 2% Conversion to open surgery
York [37]	2002	5	s-AAA	“Stacked” Technique	AneuRx cuffs	6.1	No one	No one
Ruppert [47]	2007	41	s-AAA, PAU	“Trombone” technique	Zenith (custom), Powerlink	21.9	9.8% (4/41) no aortic correlated	9.8% Endoleak: type I (1/41) type II (2/41) type III (1/41)
Jones [48]	2012	8	s-AAA, PAU	“Trombone” technique	Zenith	0.7	No one	No one
Giagtzidis [38]	2016	5/16	s-AAA	“Telescopic” technique	Endurant cuffs	12	No one	Endoleak: type II (1/5)
D’Oria [49]	2019		s-AAA	Custom-made Unibody Conical Endograft	JOTEC Extra Design Engineering stent graft	12	No one	No one
Hoshina [50]	2022	48/175	s-AAA	AFX Japan registry	AFX2	36	0.7% no aortic correlated	Endoleak: type III (3.1%)

## Data Availability

Data can be made available upon explicit request to the corresponding author.

## Abbreviations

**Complete name**	**Abbreviation**
non-infected lesions of the abdominal aorta	fl-AAA
Penetrating aortic ulcers	PAU
intramural hematoma	IMH
Saccular aneurysm	sa-AAA
Fenestrated endovascular aneurysm repair	FEVAR
abdominal aortic aneurysms	AAA
infective native aortic aneurysms	INAA
aortic dissection	AD
descending thoracic aorta	DTA
abdominal aorta	AA
European Society of Vascular Surgery	ESVS
Society for Vascular Surgery	SVS
endovascular aneurism repair	EVAR
open surgery repair	OSR
saccular lesion of abdominal aorta	sl-AAs
computed tomography angiography	CTA
complex abdominal aneurysmand	cAAA
thoracoabdominal aneurysms	TAAA
target visceral vessel	TVV
